# Nuclear Factor-kappa B as a Resistance Factor to Platinum-Based Antineoplasic Drugs

**DOI:** 10.1155/2008/576104

**Published:** 2008-04-08

**Authors:** Vilma Maldonado Lagunas, Jorge Meléndez-Zajgla

**Affiliations:** ^1^Laboratorio de Biología Molecular, Subdirección de Investigación Básica, Instituto Nacional de Cancerológica, Avenida San Fernando 22, 14080 Tlalpan, Mexico; ^2^Genómica Funcional de Cáncer de Laboratorio, Instituto Nacional de Medicina Genomita, 03020 Mexico City, Mexico

## Abstract

Platinum drugs continue to be major chemotherapy drugs for cancer treatment. Nevertheless, acquired or intrinsic resistance to these compounds is common in human tumors. One important mechanism for this resistance is the avoidance of cells entering the apoptotic pathway. Nuclear factor-kappa B (NF-kappa B, NF-*κ*B) is a pleiotropic transcription factor key in determining the death threshold of human cells. This factor is important in the final response of cells to platinum drugs, as exemplified by in vitro and in vivo models showing that inhibition of NF-*κ*B sensitizes cancer cells to the effects of these drugs. New approaches focusing on the inhibition of NF-*κ*B could help to minimize or even eliminate intrinsic or acquired resistance to platinum drugs.

## 1. INTRODUCTION

**Figure 1:**
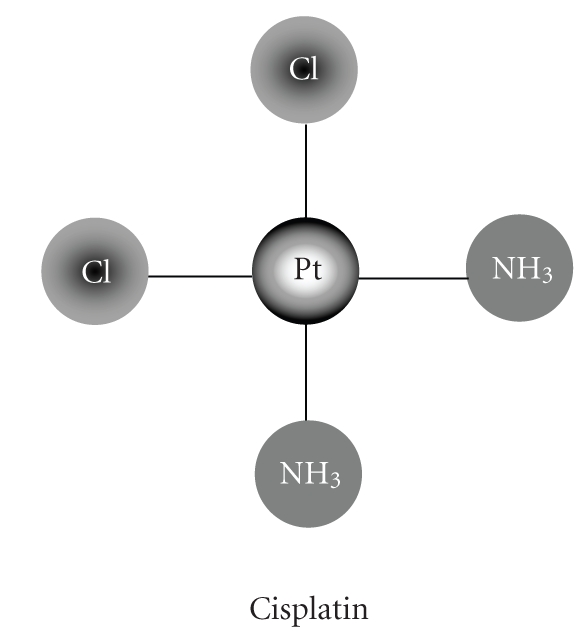
Structure of cisplatin: *cis*-diamminedichloroplatinum (II).

*Cis*-diaminedichloroplatinum (II), first known as Peyrone' salt, was synthesized in 1844 by the Italian doctor, Michele Peyrone [1]. Fifty years later, Alfred Werner “the Father of Coordination Chemistry” elucidated its structure [2]. This inorganic compound now known as cisplatin or CDDP is a neutral complex, Pt (NH_3_)_2_Cl_2_, with a central platinum atom (Pt), two chloride atoms (Cl-), and two molecules of ammonia (see Figure 1). In 1965, American chemist Rosenberg et al., in Michigan State University, found that electrolysis with platinum electrodes inhibits the growth of *Escherichia coli* bacteria. This research group determined that platinum oxidized by electrolysis to Pt^+2^ reacts with sodium chloride and ammonium salts in the bacterial growth media, forming cisplatin [3]. Due to the ability of cisplatin to inhibit cell division, Rosenberg analyzed its possible anticancer properties and found that, indeed, this compound inhibited the growth of sarcomas transplanted into rats. Nowadays, cisplatin has become one of the major chemotherapy drugs [4].

## 2. CISPLATIN MECHANISM OF ACTION

**Figure 2:**
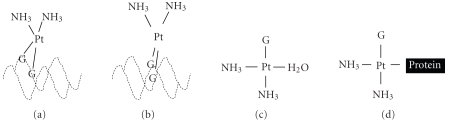
Cisplatin bound to DNA (a) intrastrand crosslink (b), interstrand crosslink (c), and monoadducts (d).

Cisplatin enters the cell mainly by passive diffusion, although its efflux and uptake have been linked to copper metabolic pathways, implicating the high-affinity cooper transporter (CTR1) and the copper-transporting P-type adenosine triphosphate (ATP-7B) [5, 6]. Once inside the cell, cisplatin forms adducts with DNA with a preference for nucleosomal regions. In this process, cisplatin losses one of its chloride ions and binds a molecule of water in order to attach to the nitrogen-7 position of a DNA purine. Subsequently, the other chloride is replaced by another molecule of water, thereby binding to DNA in a covalent form to produce 1, 2 or 1, 3 intrastrand or interstrand cross-links. Cisplatin also forms simple monoadducts with DNA, or monoadducts that bind also to proteins or glutathione molecules (see Figure 2) [7, 8]. The importance of this molecular mechanism is highlighted by the reports showing that the level of platinum-DNA adducts correlates with clinical response of cisplatin [9, 10].

DNA damage produced by cisplatin is detected and repaired by the nucleotide excision pathway (NER) [11, 12]. This pathway involves two subpathways; transcription-coupled NER and global genomic NER. Furuta et al. [12] reported that transcription-coupled NER-deficient cells are hypersensitive to cisplatin, irrespective of their global genomic NER status, showing that the former pathway could be responsible for resistance to the platinum drug. If the damage produced by cisplatin is not totally repaired, cells emit signals to initiate cellular death through apoptosis or necrosis, depending on the particular cisplatin concentration and specific tissue involved [13].

Several signal transduction pathways are activated in the cell after exposure of cisplatin, including the 3 main subfamilies of MAPK kinases, namely, extracellular signal-regulated kinase (ERK) [14], c-Jun NH2-terminal kinase (JNK) [15, 16], and p38 mitogen-activated protein kinase (p38 MAPK) [17, 18]. Cisplatin also activates v-akt murine thymoma viral oncogene homologue (AKT) [19, 20] and nuclear factor-kappa B (NF-*κ*B) pathways [21, 22].

## 3. NF-KAPPA B TRANSDUCTION PATHWAY

**Figure 3:**
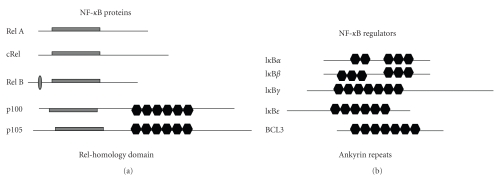
NF-*κ*B family proteins.

NF-kappa B is a family of transcription factors constituted by 15 dimers that result from different combinations of 5 proteins (Rel (cRel), Rel A (p65), Rel B, NFkB-1 (p105/p50), and NFkB-2 (p100/p52)). Each of these subunits contains a 300-amino acid Rel homology (RH) domain, which has the ability to bind to a defined DNA sequence (see Figure 3) [23]. These dimers regulate the expression of hundreds of genes involved in immune and inflammatory response, proliferation, differentiation, and cell survival. However, examples are also known where NF-*κ*B functions as a proapoptotic factor. The control over cell survival is achieved mainly through upregulation of the antiapoptotic proteins, cIAP1, cIAP2, XIAP, Blf/A1, BCL-xL, and FLIP, whereas the proapoptotic activity is mediated by FAS, FASL, DR4, and DR5 genes [24]. Although not universal, it seems that the antiapoptotic functions of NF-*κ*B are mediated by dimers containing the relA subunit of this transcriptional factor.

**Figure 4:**
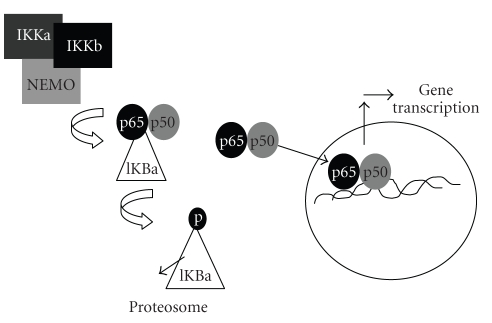
Molecular mechanisms of NF-*κ*B activation.

One critical step in the control of NF-*κ*B activity is the association of these dimers with members of the inhibitor of kappa B family (Ikappa-B alpha, Ikappa-B beta, Ikappa-B epsilon, p105/gamma, p100/delta, and BCL3). The union of a particular dimer with one Ikappa-B molecule prevents its nuclear translocation. NF-*κ*B subunits can be released from its inhibitor by specific posttranslational processes, such as phosphorylation or ubiquitination followed by proteosome-mediated proteolysis (see Figure 4).

The most studied upstream activator of NF-*κ*B is the inhibitor of kappa B kinase (IKK) complex. This complex contains two kinase catalytic subunits, IKK alpha and IKK beta, as well as a helical subunit termed IKK gamma (NEMO) which plays a critical role in the assembly of the IKK complex. Both catalytic kinase subunits are highly homologous, but are activated by different stimuli. Once activated with proinflammatory cytokines such as tumor necrosis factor alpha (TNF-*α*) or interleukin-1ß (IL-1ß), IKK ß inactivates Ikappa-B-*α*, Ikappa-B-ß, and Ikappa-B-*ε*, inducing the so-called canonic NF-*κ*B pathway, described previously. IKK-*α* is activated by more diverse stimuli, such as CD40, lymphotoxin ß, or lipopolysaccharide, which induce processing of the p52 precursor protein, p100, forming homo- or heterodimers with p50 to constitute the noncanonical NF-*κ*B pathway [25].

## 4. NF-KAPPA B AND CISPLATIN RESISTANCE

Platinum drug resistance can be mediated by several mechanisms, such as drug inactivation, cellular drug efflux, alterations in drug target, modulation of DNA repair, and evasion from apoptotic cell death [13, 26].

Due to the importance of NF-*κ*B in determining the final outcome of an apoptotic insult and the fact that most cancer cells present a constitutive activation of this transcription factor, it is not unexpected that it could be involved in resistance to platinum drugs. Earlier reports showed that cisplatin is able to induce activation of NF-*κ*B [22, 27], thereby providing a mechanism of intrinsic resistance. Furthermore, low-dose gamma irradiation induces a crossresistant phenotype in HeLa cells, which is associated with NF-*κ*B activation by a deregulation of silencer of death domain (SODD) protein expression [28]. NF-*κ*B activation after cisplatin exposure seems to be a widespread phenomenon in cancer [29] and normal cells [30]. However, cisplatin exposure results in downregulation of NF-*κ*B activity in hepatoma cells [31] although the reason for this remains unclear. Tissue-specific differences could play a role since mice lacking p65 subunit die at 15 days of gestation by massive liver cell apoptosis, showing a particular and specific requirement for the NF-*κ*B antiapoptotic function, specifically in liver homeostasis [32]. Alternatively, the well-known negative feedback mediated by Ikappa B synthesis, which downregulates NF-*κ*B activity after an initial stimulus, could explain this contradiction [33]. Further complicating this situation, different combinations of NF-*κ* subunits are known to have opposing transcriptional activities, which could help explain the contradictory results. In addition, cancer cells with cisplatin-resistant phenotypes have elevated NF-*κ*B activity [21, 34] although the molecular reason behind this activation remains obscure.

Supporting the relevance of NF-*κ*B importance in the control of apoptosis induced by cisplatin is that its inhibition by different methods sensitizes cancer cells to the drug. Genistein, a soy isoflavonoid with NF-*κ*B-inhibiting properties, potentates cisplatin effects on pancreatic cancer cells [35]. Similarly, inhibition of NF-*κ*B translocation or activation increased the efficacy of cisplatin on an in vivo model of ovarian cancer [21] and on cultured head and neck [36], prostate [37], and esophageal [38] cancer cell lines. In this regard, it is noteworthy that patients with esophageal tumors resistant to chemotherapy fail to downregulate NF-*κ*B after therapy [39].

In addition, it has been shown that NF-*κ*B may be important in acquired chemoresistance since even a transient exposure to small doses of an antineoplasic agent or radiation induces cross-resistance to cisplatin by the activation of this transcription factor [28, 40, 41].

Also of note is that a recent phase I trial showed that inhibition of NF-*κ*B with bortezomib, a proteasome inhibitor, made ovarian cancer patients more sensitive to carboplatin [42]. Similarly, preclinical studies demonstrated that the newly synthesized NF-*κ*B inhibitor, dehydroxymethylepoxyquinomicin (DHMEQ), enhanced the sensitivity of YCU-H and KB cells to cisplatin [36]. Furthermore, the importance of NF-*κ*B in resistance can be found in the blocking of its activation by an adenovirus carrying a “superrepressor” form of I-*κ*B (ad-IkappaBalpha) in cisplatin-resistant lung cancer cells, which restored their sensitivity to control levels found in sensitive cell lines [43]. These results warrant further exploration of the possible clinical use of NF-*κ*B inhibitors in patients with intrinsic or acquired platinum drug-resistant cancers.

## 5. MECHANISMS OF NF-KAPPA B ACTIVATION BY CISPLATIN

After DNA damage, several transduction cascades are activated, among them JNK and p38 [16]. Activation of JNK takes place via the MEKK1/SEK1 cascade required for cell death after platinum drug exposure [44]. MEKK1 activation drives the activation of NF-*κ*B, seen after cisplatin treatment [45], providing a basis for a possible mechanism of acquired resistance. On the other hand, Yeh et al. [46] demonstrated that the MEK/ERK pathway is one of the NF-*κ*B inhibitory circuits activated after exposure of cervical cancer cells to cisplatin. This mechanism relies on the alteration of the phosphorylation of p65 by protein phosphatase-4 [46]. These cascades activate the phosphorylation, ubiquitination, and degradation of NF-*κ*B inhibitor I-*κ*B, allowing translocation of active NF-*κ*B dimers into the nucleus [22], providing a plausible basis for intrinsic or acquired resistance, as previously discussed.

## 6. DOWNSTREAM TARGETS OF NF-KAPPA B

As mentioned above, NF-*κ*B is a pleiotropic transcription factor with target genes involved in several cellular processes. At least 20 proteins involved in the regulation of apoptosis present kappa-B consensus sites in their promoters and are actively regulated by this transcription factor [47]. Although no comprehensive study of the NF-kappa B-responsive genes involved in cisplatin resistance has been published, recent reports indicate that Bfl-1/A1 [48] and c-Myc [49] could be 2 of these genes, but clearly more investigations are needed.

## 7. NEW PLATINUM COMPOUNDS

After the initial discovery of cisplatin, several analogs have been synthesized with the purpose of improving their antineoplastic activity and reducing adverse effects such as nephrotoxicity. One of the successful analogs is carboplatin, which contains a platinum atom surrounded with two ammonia groups and two other ligands in a ring structure. Cisplatin appears to be superior to carboplatin in terms of therapeutic effectiveness for some tumors such as germ cell tumors, bladder cancer, as well as head and neck cancer, while in others (e.g., lung and ovarian cancer), their efficacies are comparable [50]. Carboplatin treatment downregulates constitutive NF-*κ*B activity and prevents nuclear retention of p65 in liver cancer [51] and glioma cell lines [52].

Oxaliplatin is another cisplatin analog that contains a platinum atom complexed with 1,2-diaminocyclohexane that has an oxalate ligand. Its spectrum of activity and mechanism of action and resistance are different from cisplatin and carboplatin [53]. Downregulation of NF-*κ*B transactivation by pharmacological inhibitors enhances oxaliplatin cytotoxicity in a panel of 4 colon adenocarcinoma cell lines [54].

Recently, a new approach has been the synthesis of water-soluble platinum complexes that can be absorbed after oral administration, such as JM216 and its metabolite JM118 [55], which have demonstrable oral antitumor activity in mice broadly equivalent to intravenously administered cisplatin and a toxicological profile similar to that of carboplatin. To date, there are no studies focusing on the activity of NF-*κ*B in relation to these compounds.

A new promising approach is the encapsulation of cisplatin in sterically stabilized, long circulating, PEGylated liposomes, such as SPI-77, which show more stability in plasma and have a longer circulation time, greater efficacy, and lower toxicity than free cisplatin. Similar to this compound is lipoplatin, which is formed from cisplatin and liposomes composed of dipalmitoyl phosphatidyl glycerol (DPPG), soy phosphatidyl choline (SPC-3), cholesterol, and methoxy-polyethylene glycol-distearoyl phosphatidylethanolamine (mPEG2000-DSPE) [56, 57]. There are no studies to date on the routes that activate these new drugs.

## 8. CONCLUSION

In order to increase the benefit of current platinum-based drugs and to direct effort to obtain improved agents, it is of great importance to understand the molecular basis of acquired and intrinsic resistance. NF-*κ*B is a key to this understanding due to its importance in determining the final cell response to platinum drugs. New approaches focusing in the inhibition of this factor could help to minimize or even eliminate resistance to platinum drugs or to provide drugs with less systemic toxicity.

## Figures and Tables

**Figure 5:**
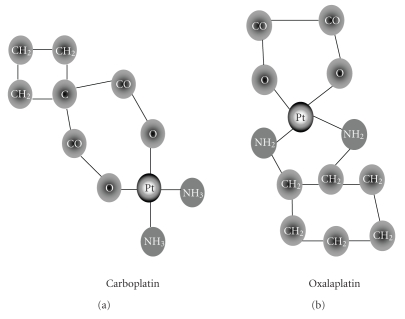
Structure of two analogs of cisplatin (a) carboplatin (b) oxaliplatin.
